# Severe tricuspid regurgitation with biannular disjunction requiring surgical treatment: a case report

**DOI:** 10.1093/ehjcr/ytae270

**Published:** 2024-05-29

**Authors:** Mayuka Masuda, Junichi Imanishi, Takeshi Inoue, Masanori Okuda

**Affiliations:** Department of Cardiology, Hyogo Prefectural Awaji Medical Center, 1-1-137, Shioya, Sumoto, Hyogo 656-0021, Japan; Department of Cardiology, Hyogo Prefectural Awaji Medical Center, 1-1-137, Shioya, Sumoto, Hyogo 656-0021, Japan; Department of Cardiovascular Surgery, Hyogo Prefectural Awaji Medicine Center, 1-1-137, Shioya, Sumoto, Hyogo 656-0021, Japan; Department of Cardiology, Hyogo Prefectural Awaji Medical Center, 1-1-137, Shioya, Sumoto, Hyogo 656-0021, Japan

**Keywords:** Tricuspid annular disjunction, Tricuspid valve prolapse, Tricuspid regurgitation, Mitral annular disjunction, Case report

## Abstract

**Background:**

Tricuspid annular disjunction (TAD) is an annular disjunction of the right-sided heart. Although TAD is often concomitant with mitral annular disjunction (MAD), it often presents as mitral regurgitation (MR), rather than tricuspid regurgitation (TR). While the clinical significance of MAD has been well-established, there is still little data on TAD. This is a rare case of severe TR due to TAD that appears to be isolated from MAD.

**Case summary:**

A 63-year-old female complaining of pre-syncope and dyspnoea on exertion was referred to our department. Initial transthoracic echocardiography showed MR and TR due to tricuspid valve prolapse (TVP). On transoesophageal echocardiography, the TVP consisted of an excessively redundant anterior leaflet, where the annular disjunction and severe regurgitation were formed. She recently underwent mitral and tricuspid valve plasties for symptomatic primary severe TR.

**Discussion:**

This case report emphasizes the clinical significance of TAD as a potential cause of severe TR, even without significant MR. Tricuspid annular disjunction progresses more gradually compared with MAD. This case suggests that remodelling of the right atrium, particularly in chronic atrial fibrillation, may contribute to the development of TR. Despite diagnostic challenges due to the flexible and dynamic nature of the tricuspid annulus, this is the first report of TAD-induced severe TR necessitating surgical intervention. Accurately diagnosing TAD remains challenging with current imaging modalities, emphasizing the need for improved diagnostic tools to optimize treatment strategies.

**Learning points**

- While the clinical significance of mitral annulus disjunction is well-established, there is still little data on tricuspid annular disjunction (TAD).
- The present case is the first report of severe tricuspid regurgitation (TR) with TAD requiring surgery, and the presence of TAD could be considered one of the aetiologies for TR.
- Most cases of TAD require cardiac magnetic resonance for diagnosis, but transthoracic echocardiography can useful in particular types of TAD.

## Introduction

The characteristics of tricuspid valve prolapse (TVP) on transthoracic echocardiography (TTE) are not well-defined. Further studies on the definition and clinical significance of TVP are needed, especially, in the light of research on tricuspid valve interventions.^1^ Mitral annulus disjunction (MAD) is a structural abnormality with an obvious separation between the mitral valve annulus and the left ventricle (LV) basal myocardium at end-systole, often accompanying mitral valve prolapse.^2^ On the other hand, tricuspid annular disjunction (TAD) refers to an annular disjunction of the right-sided heart, analogous to MAD. Although a recent study reported that TAD is often accompanied by MAD, mitral regurgitation (MR) is often highlighted instead of tricuspid regurgitation (TR).^3^ While the clinical significance of MAD is well-established, there is still little data on TAD. Herein, we present a case of severe TR due to TAD requiring surgical treatment.

## Summary figure

**Table:** 

20 months before surgery	First visit for pre-syncope and dyspnoea on exertion Initial transthoracic echocardiography: mild mitral regurgitation (MR) and severe tricuspid regurgitation (TR) due to tricuspid valve prolapse (TVP) Both MR and TR involved annular disjunction
2 weeks after the first visit	Transoesophageal echocardiography: TVP due to an excessively redundant anterior leaflet forming annular disjunction and severe regurgitation
1 year after the first visit	For symptomatic primary severe TR, surgery was performed Intervention: mitral and tricuspid valve plasty.
Post-operative course	No recurrence of heart failure

## Case presentation

**Figure 1:**
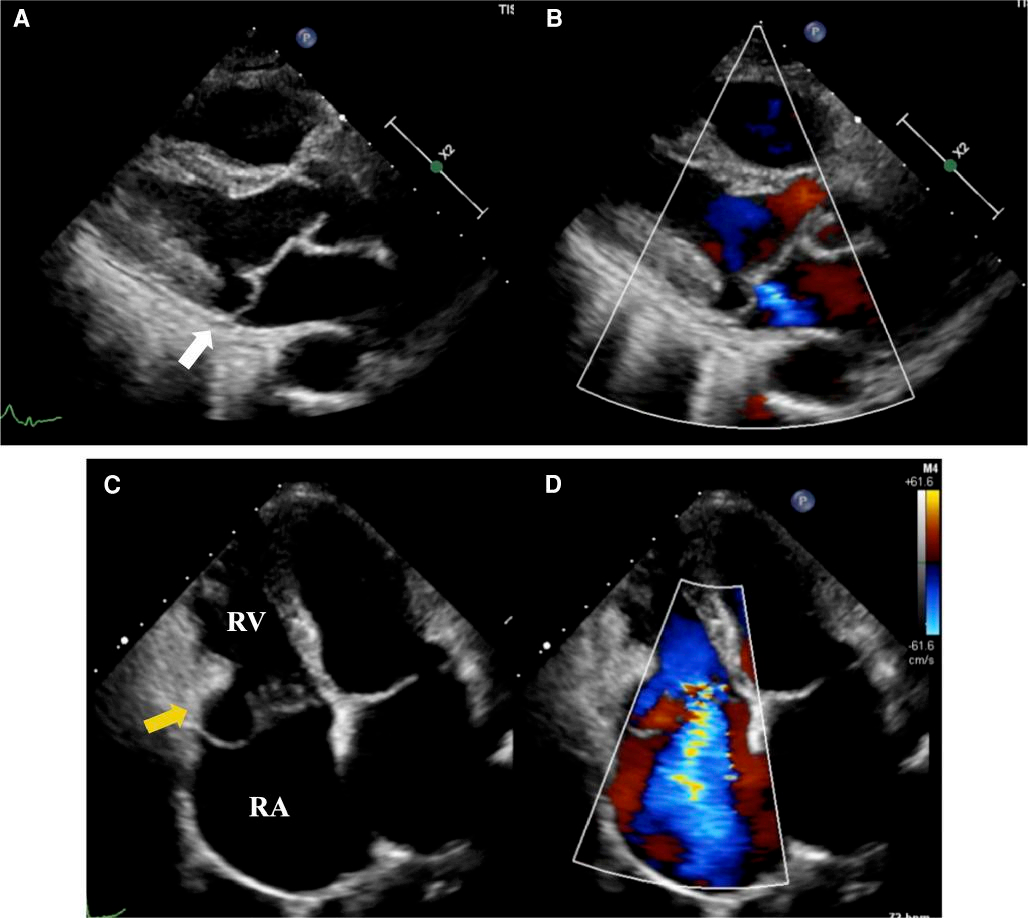
Transthoracic echocardiography images of the patient before surgery. Mitral annular disjunction in the posterolateral wall (white arrow). (*B*) Mild mitral regurgitation. (*C*) Tricuspid annular disjunction in the right ventricular free wall (yellow arrow). (*D*) Severe tricuspid regurgitation. The yellow arrows indicate areas of mitral annular disjunction. RV, right ventricle; RA, right atrium.

**Figure 2:**
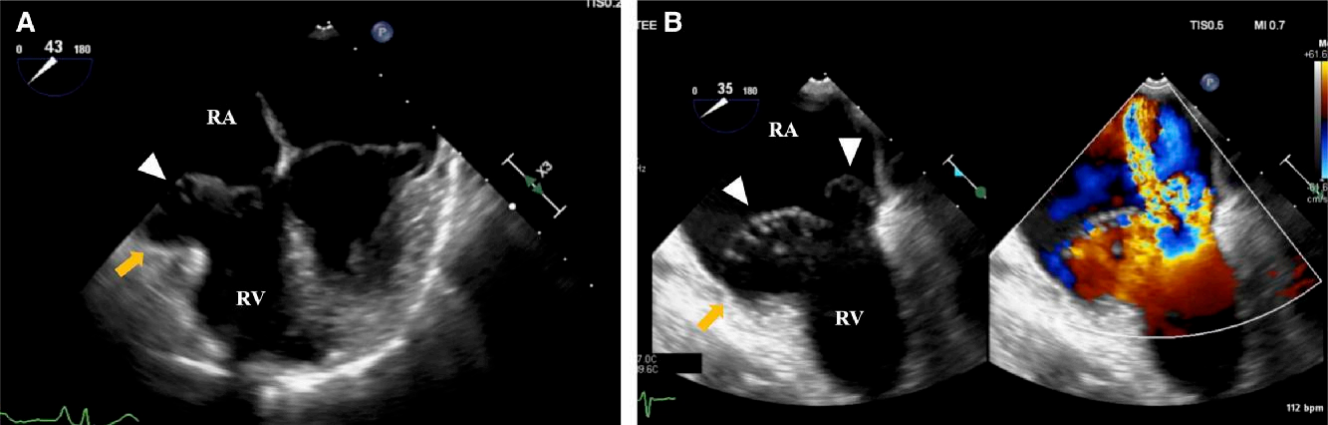
(*A*) Transoesophageal echocardiography showing mitral annular disjunction (yellow arrow) in the right ventricular free wall and prolapsed tricuspid leaflet (white arrow head). (*B*) Severe tricuspid regurgitation with a grossly redundant tricuspid valve (white arrow heads). RV, right ventricular; RA, right atrium.

**Figure 3:**
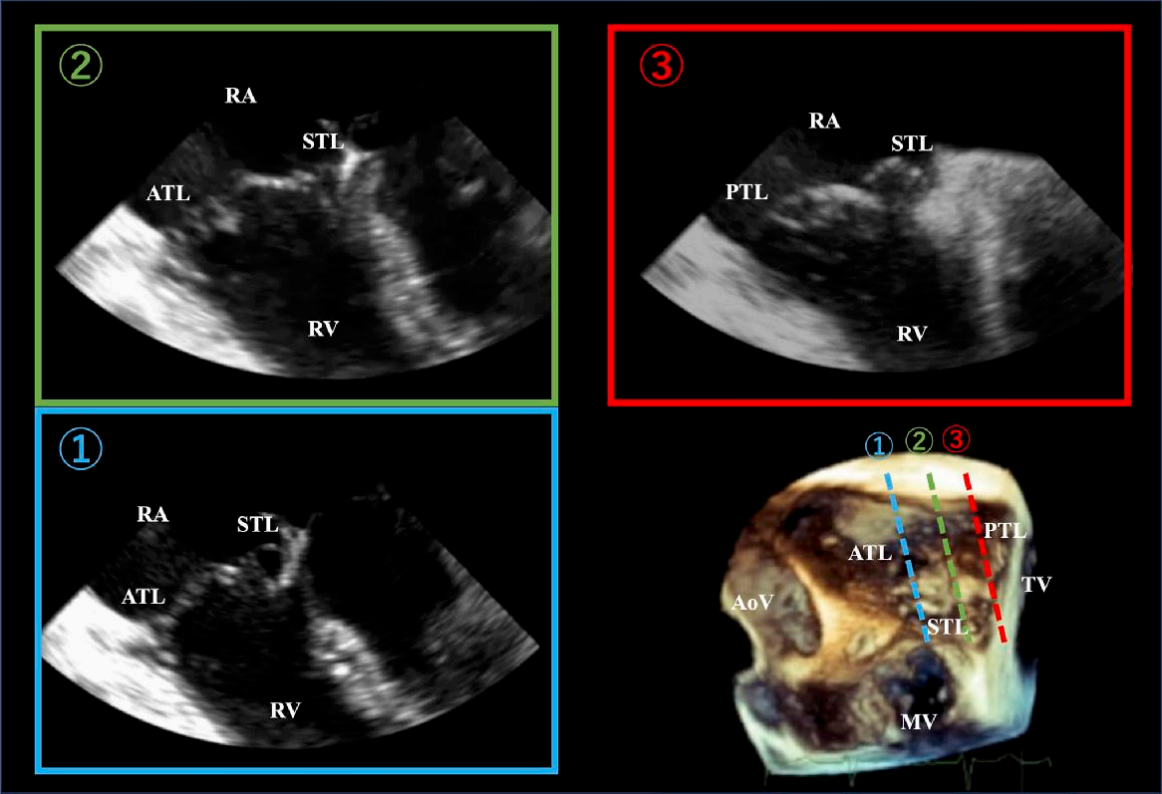
Three-dimensional multiplanar reconstruction planes through the tricuspid valve in end-systole. The three long-axis planes, which perpendicularly cross the trisecting lines to the tricuspid valve, were generated carefully using guidance on the short-axis image of the tricuspid valve.

A 63-year-old woman consulted with a primary care doctor for pre-syncope and dyspnoea on exertion. Atrial fibrillation (AF) was documented at the first visit, thus warranting referral to our department for further management. Her medical history was unremarkable. Vital signs on referral were as follows: heart rate of 58 b.p.m., blood pressure of 139/106 mmHg, and oxygen saturation of 99% on room air. The physical examination was otherwise unremarkable, except for an apical systolic murmur. Laboratory results showed normal levels of platelet (15.2 × 10^4^/μL), prothrombin time-international normalized ratio (PT-INR) (1.02), creatinine (0.7 mg/dL), estimated glomerular filtration rate (64.5 mL/min/1.73 m^2^), and increased brain natriuretic peptide levels at 145.9 pg/mL (Table 1). Electrocardiography revealed AF. Initial TTE showed a normal-sized LV with an LV ejection fraction of 61%, normal wall motion, and normal left atrial volume index (32.8 mL/m^2^). There was mild MR and severe TR due to TVP. Furthermore, both MR and TR had annular disjunction (*Figure 1*, Supplementary material online, *Video S1*). Further workup with transoesophageal echocardiography (TEE) revealed that the mitral and tricuspid valve morphologies were similar to Barlow’s disease–characterized redundant leaflets. With regard to the mitral valve, the prolapse of the bileaflet with MAD caused mild MR. On the other hand, the TVP consisted of an excessively redundant anterior leaflet, where the annular disjunction and severe regurgitation developed (*Figure 2*, Supplementary material online, *Video S2*). The three-dimensional multiplanar reconstruction planes through the TV in end-systole showed that each leaflet of the tricuspid valve was redundant and prolapsed (*Figure 3*). Transthoracic echocardiography and TEE confirmed severe TR, as measured by vena contracta of 12 mm and TR jet area of 13.2 cm^2^ (50.2%). The right chambers were significantly enlarged [right atrial indexed volume area 26.3 cm^2^; right ventricular (RV) basal diameter 43.6 mm], whereas TV annulus was dilated (40.3 mm). Furthermore, RV function was preserved [tricuspid annular plain systolic excursion 23.2 mm, fractional area change 39%, lateral wall tissue Doppler 12.5 cm/s]. The estimated RV systolic pressure was 31 mmHg. Cardiovascular magnetic resonance imaging showed no myocardial late gadolinium enhancement. The 24 h Holter revealed a rhythm of AF throughout, with a small number of premature ventricular contractions (0.07% of total beats), which were polymorphic but mainly monomorphic.

**Table 1:** Laboratory findings on referral

Laboratory test	Patient’s value	Reference ranges
White blood cell count	5.8	3.3–8.6 × 10^3^/μL
Haemoglobin	14.1	11.6–14.8 g/dL
Platelet	15.2 × 10^4^	15–40 × 10^4^/µL
Sodium	141	138–145 mmol/L
Potassium	4.3	3.6–4.8 mmol/L
Chloride	107	101–108 mmol/L
Albumin	3.8	4.1–5.1 g/dL
Blood urea nitrogen	10.9	8–20 mg/dL
Creatinine	0.7	0.46–0.79 mg/dL
Estimated glomerular filtration rate	64.5	≥ 60 mL/min/1.73 m^2^
Total bilirubin	0.74	0.4–1.5 mg/dL
AST	40	13–30 U/L
ALT	38	7–23 U/L
LD	165	124–222 U/L
CRP	0.04	0.00–0.14 mg/dL
BNP	145.9	<18.4 pg/mL

ALT, alanine aminotransferase; AST, aspartate aminotransferase; BNP, brain natriuretic peptide; CRP, C-reactive protein; LD, lactate dehydrogenase.

Surgery was recommended because of the symptomatic primary severe TR. Since the patient was relatively young at the age of 63, we decided that a complete control of the TR by surgery was desirable. Pre-operative TEE of the mitral valve revealed a Barlow-like redundant leaflet with MAD and an extensive prolapse of both leaflets. Although regurgitation was only mild, there was concern that prolapse and regurgitation might develop in the future due to the closing force during the closure of the redundant valve leaflet. Therefore, we decided to intervene on the mitral valve as well. However, the patient refused immediate surgery, and optimal medication therapy (bisoprolol fumarate, 0.625 mg; azosemide, 15 mg; furosemide, 10 mg; apixaban, 10 mg) was initiated. The patient was observed on an outpatient basis for a year after the initial examination, but dyspnoea on exertion gradually worsened. Thus, surgery was planned. There were no significant findings on pre-operative investigation, including coronary angiography, and neither MR nor TR severity changed from the initial assessment.

**Figure 4:**
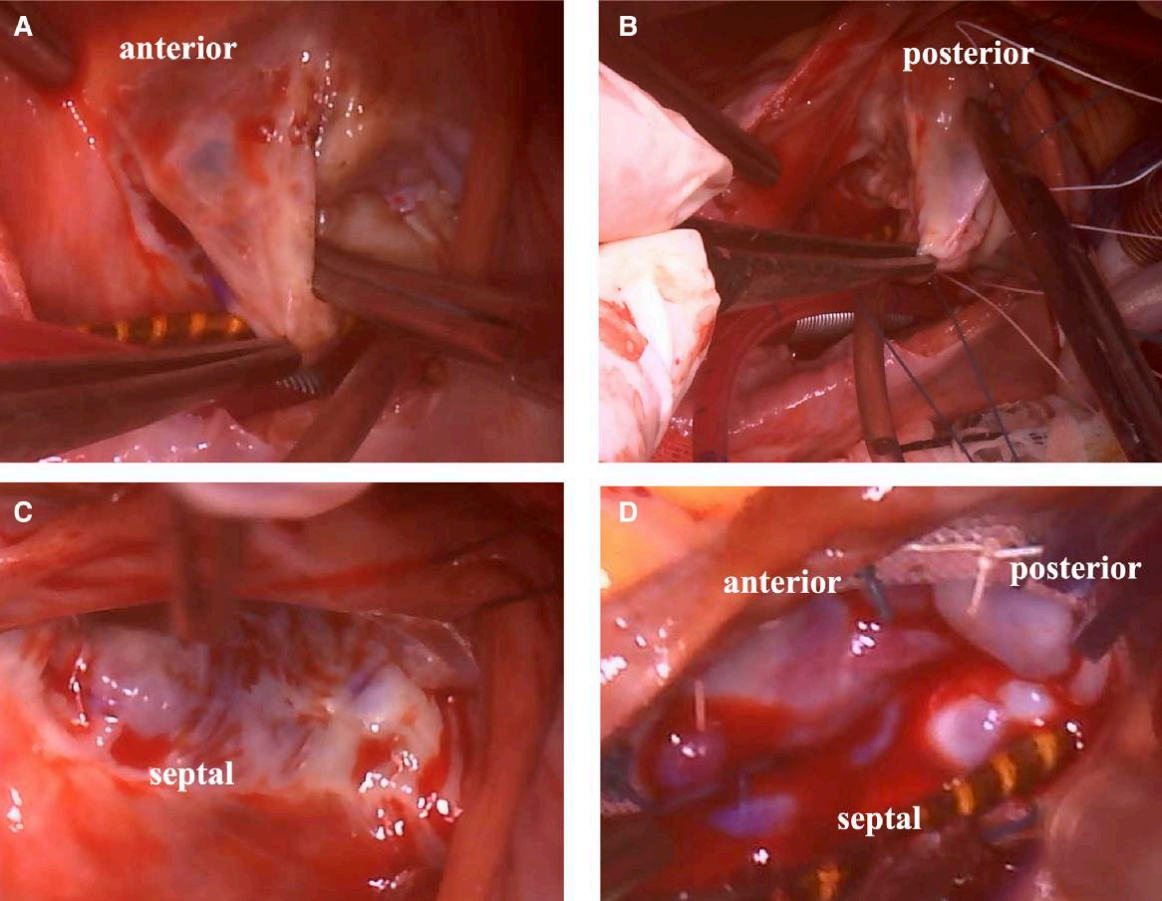
Intra-operative findings seen from the surgeon’s view: (*A*, *B*) A billowing of the posterior and anterior leaflets of the tricuspid valve, which appeared bulky and thick. (*C*) The septal leaflet of the tricuspid valve. (*D*) A saline test performed after tricuspid valve repair.

Intra-operative examination revealed a redundant mitral valve with prolapse, as well as a myxomatous and prolapsed tricuspid valve with the anterior and posterior leaflet (*Figure 4*). A pair of artificial chordae was reconstructed from the anterior and posterior papillary muscles to A2 and P2 for mitral valve repair. An annuloplasty ring (SimuPlus 32 mm) was sutured to the mitral valve. For the tricuspid valve, an annuloplasty band (Physio tricuspid 28 mm) was sutured, and a pair of artificial chordae was built attached to the anterior and posterior leaflets. These procedures improved the regurgitation of both valves. Transthoracic echocardiography on post-operative Day 6 showed an ejection fraction of 60%, trivial MR, and trivial TR without pulmonary hypertension. The post-operative course was uneventful, and the patient was discharged on Day 11 post-operatively.

## Discussion

Two clinical issues are seen in this case. First, TAD can be an important cause of severe TR without significant MR. Second, TTE may be useful to detect TAD-induced TR. In a recent study, TAD was observed in 42 out of 84 (50%) patients with MAD.^3^ In contrast, advanced changes in leaflet and annular disjunction are frequently observed in the left-sided heart. This is because annular disjunction progresses with age, whereas the tricuspid annulus is spared from haemodynamic stress because it belongs to the right-sided heart. Consequently, TAD progresses more gradually than MAD. Although both TAD and MAD were present in this case, regurgitation was more severe in the tricuspid valve, which has two possible explanations. First, the less-developed fibrous skeleton in the tricuspid annulus may lead to excessive remodelling in the right-sided heart.^4^ Such remodelling of the right-sided heart is widely seen in isolated TR, wherein AF is closely involved in its aetiology. In a study comparing functional TR caused by chronic AF without left-sided heart disease (AF-TR) vs. functional TR caused by left-sided heart disease with sinus rhythm (LH-TR), the AF-TR group had greater right atrial dilatation and a higher ratio of right-to-left atrial volume than the LH-TR group.^5^ In the AF-TR group of their study, the right atrial volume index was larger at 50.6 (36.9–71.7) mL/m^2^, whereas the left atrial volume index was smaller at 38.0 (32.5–49.9) mL/m^2^. Thus, when chronic AF occurs without significant valvular disease, it is possible that more progressive remodelling is occurring in the right atrium vs. the left atrium. Consequently, it is assumed that the subsequently enlarged tricuspid annulus leads to TR. Second, TVP due to a redundant anterior leaflet may create an environment wherein the right atrium is vulnerable to capacitive and pressure loads. Therefore, TVP and chronic AF may expedite a remodelling of the right atrium. In addition, the reduced cardiac output of the right atrium reduces the left atrial preload, leading to more excessive remodelling in the right atrium. Thus, a more conspicuous regurgitation and prolapse were observed in the right-sided heart.

It is more difficult to diagnose TAD than MAD because of the structural difference between the mitral and the tricuspid annuli. The tricuspid annulus is more flexible and dynamic because of its lower fibrotic skeleton.^4^ Tricuspid leaflets are also thinner than mitral leaflets. Since TAD is difficult to diagnose with the usual imaging modalities, cardiac magnetic resonance imaging clinches the diagnosis in many cases. Transthoracic echocardiography is unable to diagnose TAD because TTE can focus only on a limited part of the tricuspid annulus, and thus, its diagnostic ability depends on leaflets and the site of annular disjunction. Because the optimal view for observing the posterior leaflets is limited to the long-axis plane of RV inflow,^6^ a detailed observation of the posterior leaflets is generally considered challenging. In a previous analysis on the location of TAD, 16 out of 42 (38%) patients had TAD in the lateral RV free wall only, 7 (17%) had TAD in the inferior RV free wall only, and 19 (45%) had TAD in both locations.^3^ In this case, the TAD was located at the back of the anterior leaflet in the lateral free wall, allowing its diagnosis via TTE during the initial examination.

A few case reports have reported an association between TAD and ventricular arrhythmias,^7^ potentially through mechanisms similar to those observed in mitral valve pathologies. However, other evidence suggests that TAD is not associated with an increased risk of ventricular arrhythmic events.^3^ Currently, there is no evidence to suggest an association between TAD itself and AF, and we consider that it may be a secondary change caused by TR.

This is the first report of TAD-induced severe TR requiring surgical treatment. A detailed evaluation of TAD remains challenging with current imaging modalities, but it could allow us to provide the optimal treatment.

## Supplementary Material

ytae270_Supplementary_Data

## Data Availability

The data underlying this article will be shared on reasonable request by the corresponding author.

## Lead author biography

Mayuka Masuda has been a clinical trainee at the Department of Cardiology, Hyogo Prefectural Awaji Medical Center, since 2021.

## Supplementary material

**Consent:** The authors confirm that written consent for the submission and publication of this case report, including images and associated text, has been obtained from the patient in line with COPE guidance.

**Funding:** None declared.
